# Sexual reproductive health, NCDs and infectious diseases

**DOI:** 10.4314/ahs.v22i1.1

**Published:** 2022-03

**Authors:** James K Tumwine

**Affiliations:** Editor in Chief, African Health Sciences

I am pleased to welcome you to this 2022 first issue of African Health Sciences covering reproductive health, non-communicable diseases and infections. Because of the COVID-19 pandemic we received unprecedented high numbers of manuscripts and a corresponding proportion of accepted papers for publication. This, inevitably, put a lot of strain on our volunteers and hence the slight delay in releasing this issue.

In the reproductive health section, we bring you 24 papers on condom use and family planning, 1–4 sexuality^5^–^8^ and menstruation 9,10. This is followed by papers on cancer and other neoplasms in women 11–16. Then comes the section on pregnancy and its outcomes and complications 17–23, as well as the importance of midwifery^24^.

We follow this up with non-communicable diseases. This section is diverse and includes articles on the cardiovascular system 25–27 such as cardiomyopathy, arrhythmias in adults and pulmonary hypertension in children. It also includes substance abuse, other mental health and neurological diseases 28–33. We touch on metabolic disorders and cancer^34^–^41^, as well as traditional herbs, genes and trauma^42^–^48^.

We have a major section on infectious diseases and these are dominated by HIV^49^–^55^ and other infections such as hepatitis B, tuberculosis, bacterial infections, Covid-19 and others^56^–^69^.

The rest of the first issue of this 22^nd^ volume of African Health Sciences ranges from disability, surgery, to haematology and training^70^–^79^. Finally, we wish to thank all our partners, readers, volunteers, editorial and board teams, authors and reviewers. Thank you very much for your support as we strive for excellence in publishing for better health in Africa and the diaspora.
